# The effects of pravastatin on the normal human placenta: Lessons from *ex-vivo* models

**DOI:** 10.1371/journal.pone.0172174

**Published:** 2017-02-15

**Authors:** Adelina Balan, Irit Szaingurten-Solodkin, Shani S. Swissa, Valeria Feinshtein, Mahmoud Huleihel, Gershon Holcberg, Doron Dukler, Ofer Beharier

**Affiliations:** 1 The Shraga Segal Department of Microbiology and Immunology, Faculty of Health Sciences, Ben-Gurion University of the Negev, Beer-Sheva, Israel; 2 Department of Physiology and Cell Biology, Faculty of Health Sciences, Ben-Gurion University of the Negev, Beer-Sheva, Israel; 3 Department of Clinical Biochemistry and Pharmacology, Faculty of Health Sciences, Ben-Gurion University of the Negev, Beer-Sheva, Israel; 4 Department of Obstetrics and Gynecology, Soroka University Medical Center and Ben-Gurion University of the Negev, Beer Sheva, Israel; Otto von Guericke Universitat Magdeburg, GERMANY

## Abstract

**Introduction:**

Research in animal models and preliminary clinical studies in humans support the use of pravastatin for the prevention of preeclampsia. However, its use during pregnancy is still controversial due to limited data about its effect on the human placenta and fetus.

**Methods:**

In the present study, human placental cotyledons were perfused in the absence or presence of pravastatin in the maternal reservoir (PraM). In addition, placental explants were treated with pravastatin for 5, 24 and 72 h under normoxia and hypoxia. We monitored the secretion of placental growth factor (PlGF), soluble fms-like tyrosine kinase-1 (sFlt-1), soluble endoglin (sEng), endothelial nitric oxide synthase (eNOS) expression and activation and the fetal vasoconstriction response to angiotensin-II.

**Results:**

The concentrations of PlGF, sFlt-1 and sEng were not significantly altered by pravastatin in PraM cotyledons and in placental explants compared to control. Under hypoxic conditions, pravastatin decreased sFlt-1 concentrations. eNOS expression was significantly increased in PraM cotyledons but not in pravastatin-treated placental explants cultured under normoxia or hypoxia. eNOS phosphorylation was not significantly affected by pravastatin. The feto-placental vascular tone and the fetal vasoconstriction response to angiotensin-II, did not change following exposure of the maternal circulation to pravastatin.

**Conclusion:**

We found that pravastatin does not alter the essential physiological functions of the placenta investigated in the study. The relevance of the study lays in the fact that it expands the current knowledge obtained thus far regarding the effect of the drug on the normal human placenta. This data is reassuring and important for clinicians that consider the treatment of high-risk patients with pravastatin, a treatment that exposes some normal pregnancies to the drug.

## Introduction

Preeclampsia complicates 3–8% of pregnancies and can cause multisystem maternal organ injury [1, 2]. To date, preeclampsia remains incurable and delivery is the only way to stop disease progression [3]. When clinical preeclampsia develops at early gestation, delivery may result in severe neonatal prematurity. Thus, despite excessive research effort, the disease is still a leading cause of maternal and neonatal morbidity and mortality [3].

Preeclampsia begins early in pregnancy and is characterized by an abnormal invasion of trophoblasts and impaired re-demodulation of the uterine spiral arteries [1, 2]. These events induce oxidative stress and hypoxic injury in the placenta, ultimately leading to an angiogenic imbalance characterized by extensive secretion of the anti-angiogenic factors soluble fms-like tyrosine kinase 1 (sFlt-1) and soluble endoglin (sEng) [1, 2]. Elevated levels of the anti-angiogenic factors antagonize the effects of the pro-angiogenic factors, vascular endothelial growth factor (VEGF) and placental growth factor (PlGF) leading to widespread endothelial dysfunction [4–6] and the clinical features of preeclampsia [1, 2].

Statins are among the most extensively investigated and prescribed medication in clinical use. Based on their cholesterol-lowering properties, statins are successfully used for the prevention of cardiovascular diseases [7]. Additionally, substantial evidence supports the notion that statins may reduce tissue damage through cholesterol- independent pathways named—pleiotropic effects [8]. These pleiotropic properties, including: pro-angiogenic, anti-inflammatory, anti-oxidant and anti-thrombotic, contribute to endothelial protection [9–11]. Since preeclampsia is associated with widespread endothelial damage, the protective pleiotropic effects of statins on the endothelium, mark them as a potential therapeutic option for the treatment of preeclampsia [12, 13]. Indeed, evidence obtained from different animal models demonstrates that statins could abolish the clinical phenotype of preeclampsia and restore angiogenic balance [14–18]. Recently, the first randomized pilot clinical study in humans was published [19]. In this study, a small group of 20 high-risk patients were treated with pravastatin versus placebo during pregnancy. While 40% of the placebo patients developed preeclampsia with an imbalanced angiogenic state, patients that received pravastatin did not develop the disease and maintained a normal angiogenic balance [19]. In addition, pravastatin treatment was found to improve pregnancy outcomes of women with refractory obstetric antiphospholipid syndrome when taken at the onset of preeclampsia or severe IUGR [20]. Despite these encouraging results, our knowledge regarding the effect of the drug on the human placenta and feto-placental circulation is extremely limited. In this regard, caution should be taken when considering the use of pravastatin in high-risk pregnant women, since the treatment may expose some women with normal pregnancy to the drug.

The major concern of using statins during pregnancy is fetal exposure, as it can potentially lead to fetal malformations due to inhibition of cholesterol synthesis. This concern was previously addressed using the *ex-vivo* human perfused cotyledon model that indicated limited transfer of the drug via the placental barrier [21, 22]. Moreover, the concentration of pravastatin in the umbilical cord after delivery following maternal treatment was found to be below the limit of detection [19]. These reports indicate that fetal exposure following maternal treatment is minimal. Regarding the potential effect of the drug on the normal pregnancy, two different mechanisms justify further investigation:**1.** The effect of pravastatin on placental angiogenic balance. A large amount of data from animal models demonstrates that pravastatin can prevent the angiogenic imbalance that characterizes preeclampsia [15–18]. However, this potent effect, that may be utilized to prevent preeclampsia, might theoretically have negative effects on the normal placenta. **2.** The effect of pravastatin on the fetal vasoconstriction response to acute hypoxia. Recent studies mark the endothelial nitric oxide synthase (eNOS)-nitric oxide (NO) axis as a crucial pathway in placenta-related pathologies. In these studies Sildenafil treatment, that specifically increase NO concentrations, improved the outcome of pregnancies complicated by IUGR and preeclampsia [23–25]. Although pravastatin was found to activate eNOS in endothelial cells independently of its cholesterol-lowering actions [26] its effect on placental eNOS requires further investigation. One of the most common challenges to the fetus during gestation is a reduction in oxygenation or acute hypoxia [27]. The fetal cardiovascular response to such episodes includes redistribution of the cardiac output away from peripheral circulation towards essential organs by peripheral vasoconstriction. This essential defense mechanism allows adequate perfusion of the brain [28, 29] and is triggered by a chemoreflex and maintained by constrictor hormones [29, 30]. NO synthesis during hypoxia opposes these mechanisms, however the balance of all effects favors constriction [31]. Reports from the study of Kane *et al*. in sheep model question the potential safety profile of pravastatin [32], due to its ability to attenuate the fetal peripheral vasoconstriction responses to acute hypoxic stress [32]. This effect was reported to be mediated via increased NO bioavailability [32]. This issue was not addressed in human culture or tissue until now.

Two main models exist for the study of the human placenta: the human placental perfused cotyledon model and villous placental explant cultures. The human placental perfused cotyledon model most closely resembles the *in vivo* situation as structural integrity and cell—cell organization are maintained [33]. However, its limitation lays in the fact that approximately 80% of the placentas have fetal leak and are disqualified from the experiment, resulting in low successful perfusion rate [33]. In addition, the use of this model limit the experiment to only a few hours as fetal leakage and the resistance in the feto-placental vasculature are occasionally increased in prolonged perfusion [33]. Villous placental explant cultures have the advantage of retaining the spatial relationships between the various cell types of the villous and allow conducting experiments for up to several days. Thus, a combination of the two models helps to overcome the limitations.

In the present study we used the human placental perfused cotyledon *ex-vivo* model and cultured placental explants under normoxia and reduced O_2_ conditions. We investigated the effect of pravastatin on the secretion of angiogenic factors, the expression and activation of eNOS, the secretion of NO from the placenta, the feto-placental vascular tone and the fetal vasoconstriction response to Angiotensin-II.

## Materials and methods

### Human placental perfused cotyledon ex-vivo model

Placentas from uncomplicated term (37–41 weeks) pregnancies were obtained immediately after vaginal delivery and after a signed informed consent was given. Inclusion criteria included no maternal infections, no meconium stain or systemic diseases and singleton pregnancy. The study was approved by Soroka university medical center institutional review board (project # 0080-14-SOR). Perfusion experiments were performed using the method of Schneider *et al*. [34, 35].

A fetal artery-vein pair was cannulated while assuring appropriate pressure (20–60 mm Hg) using an internal pressure monitor (series 50 IP-2; Philips, Ontario, Canada). Maternal flow was established with blunt tipped needles inserted into the maternal tissue at the fetal pair site. Residual blood was removed by a pre-perfusion period of 30 minutes with medium 199 (Sigma Aldrich, Rehovot, Israel) containing albumin (3 g/L), heparin (2000 U/L), gentamicin (40 mg/L) and glucose (1 g/L). The circuits were then closed, perfusates were refreshed and a 5 h perfusion experimental period began. Perfusates were equilibrated with a pre-humidified gas mixture of 95% O_2_ and 5% CO_2_ in the maternal reservoir and 95% N_2_ and 5% CO_2_ in the fetal reservoir. HCl was used to retain fetal and maternal pH at 7.25–7.35 and 7.35–7.45, respectively. Placental viability was ensured by monitoring feto-placental pressure, pH, fetal reservoir volume, glucose and lactate concentrations (S1 Table) and the contraction response to 100 nM angiotensin II.

### Experimental protocol

Isolated placental cotyledons were perfused in the absence (Cntl) or presence of 0.2 micromol/L (100 ng/ml; Twice the serum concentration of a 40 mg daily dose) pravastatin (Sigma-Aldrich Israel, Rehovot, Israel) in the maternal circulation only (PraM). The secretion of angiogenic factors was measured by ELISA (R&D Systems, Minneapolis, MN, USA)

### Culture of placental explants

Human placental tissue was collected from normotensive pregnancies immediately after vaginal delivery and after a written informed consent was given. A piece of 5–6 g of cotyledon was excised at random and rinsed extensively in sterile phosphate buffered saline (PBS; Biological Industries, Israel) to remove blood. Decidua and large vessels were removed by blunt dissection. Villous tissue was then finely dissected into 5–10 mg pieces while in an iced sterile PBS bath. The pieces were washed two more times before culture. Fifty milligrams of villous tissue was placed into each well of a 24 well plate (Greiner Bio-One GmbH, Kremsmünster, Austria) containing 1.0 ml of Medium 199 supplemented with 10% Fetal Bovine Serum (Biological Industries, Israel) and antibiotics. Explants were incubated at 37°C for a 24 h pre-incubation period on an orbital shaker (60 rpm, Flask Dancer, Boekel Scientific, PA, USA) under standard tissue culture conditions of 5% CO_2_-balanced room air (normoxia; 21% O_2_). After medium change and the addition of pravastatin, the plates were placed at 37°C for different time durations in either normoxia or reduced O_2_ (hypoxia; 1% O_2_-5% CO_2_-94% N_2_).

### Western blotting

Tissue samples were homogenized in lysis buffer containing 210 mM manitol, 70 mM sucrose, 10 mM Tris pH 7.4, 2% Triton-X100, 1 mM EDTA, protease (Sigma-Aldrich Israel, Rehovot, Israel) and phosphatase inhibitor (Santa Cruz Biotechnology, INC., USA) cocktails. Total lysis was confirmed by sonication. Samples were subjected to SDS-PAGE. The expression of eNOS and GAPDH was determined by rabbit polyclonal antibodies from Santa Cruz Biotechnology, INC., CA, USA [Working dilutions: eNOS (sc-654) 1:200; GAPDH (sc-25778) 1:5000]. eNOS activation was detected by eNOS phospho-ser 1177 rabbit monoclonal antibody (Cell Signaling Technology, Inc., MA, USA; working dilution 1:1000; cat #9570). Detection of immunoreactive bands was carried out using enhanced chemiluminescence (Biological Industries, Israel). The intensity of the bands was measured using ImageJ software (NIH, Bethesda, MD, USA).

### Measurement of NO production

The concentrations of NO were determined by measurements of its stable end product, nitrite, using Griess reagent (Sigma-Aldrich Israel, Rehovot, Israel). Concentrations were determined versus a sodium nitrite standard (0.78–50 micromol/L).

### Angiotensin-II-induced vasoconstriction

Baseline feto-placental vasoconstriction was induced by a bolus injection of 100 nM angiotensin-II (Sigma Aldrich, Rehovot, Israel). Changes in fetal perfusion pressure were recorded and the cotyledon was allowed to recover until perfusion pressure returned to baseline levels. By the end of the recovery period the circuits were closed, fetal and maternal perfusates were refreshed and a 5 h perfusion experimental period began as described above. Two consecutive vasoconstrictions were then induced by a bolus injection of 100 nM and 1000 nM angiotensin-II. Changes in fetal perfusion pressure were recorded. The cotyledon was allowed to recover between injections until perfusion pressure returned to baseline levels.

### Statistical analysis

Continuous parameters were expressed as mean ± SEM and examined using the Student’s t-test. Comparisons between experimental treatments were performed using 1-way analysis of variance followed by Tukey's posthoc analysis. *p*<0.05 was considered statistically significant.

## Results

### The effect of pravastatin on the concentrations of PlGF, sFlt-1 and sEng

We used the human placental perfused cotyledon model to study the effect of pravastatin on the secretion of angiogenic factors. Table 1 summarizes the clinical characteristics of the pregnancies. There were no significant statistical differences in maternal age, gestational age, placental weight, blood pressure, umbilical pH and the newborn weight between the experimental groups. Viability measures were within normal ranges (S1 Table).

**Table 1:** Clinical characteristics of pregnancies.

Characteristics	Cntl (n = 8)	PraM (n = 11)	P value
Gravidity, (median) ^a^	3.63±0.65 (4)	3.82±1.06 (3)	NS
Parity, (median) ^a^	2.00±0.42 (2.5)	2.27±0.80 (1)	NS
Maternal age (Yr) ^a^	26.3±1.1	26.1±2.0	NS
Gestational age (Wk) ^a^	40.0±0.4	39.8±0.3	NS
Prenatal medications	Venofer, n = 2	Metformin, n = 1	----
Smoking/alcohol	n = 1/None	None/None	----
Gestational diabetes Mellitus	None	GDM A2, n = 1	----
placental weight (g) ^a^	490.1±29.8	458.1±23.4	NS
Systolic BP (mmHg) ^a^	116.0±3.9	115.0±3.1	NS
Diastolic BP (mmHg) ^a^	67.7±5.6	63.9±3.8	NS
Umbilical pH ^a^	7.26±0.03	7.35±0.02	NS
Newborn weight (Kg) ^a^	3.2±0.1	3.2±0.2	NS

^*a*^Values are the mean ±SEM.

NS, Not significant

#### Maternal circulation

**Fig 1:**
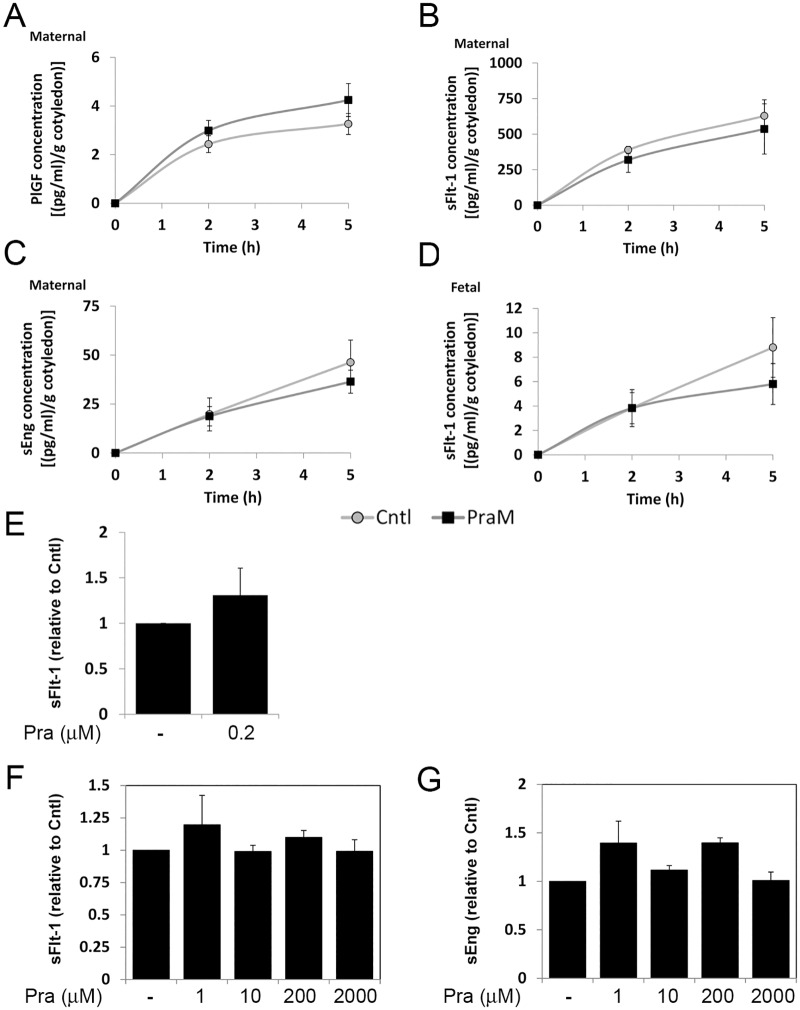
The effect of pravastatin on the release of angiogenic factors from the human placenta. Maternal concentrations of placental growth factor (PlGF; A), soluble fms-like tyrosine kinase 1 (sFlt-1; B) and soluble endoglin (sEng; C) and Fetal concentrations of sFlt-1 (D) were determined in samples collected from the maternal and fetal reservoirs of cotyledons perfused in the absence (Cntl) or presence of 0.2 micromol/L pravastatin in the maternal circulation only (PraM) as described in the "materials and methods" section. The concentrations of the different factors were normalized to gram (g) of perfused cotyledon. Cntl: n = 8; PraM: n = 11. E. The concentrations of sFlt-1 were determined in medium samples collected from placental explants incubated in the absence or presence of 0.2 micromol/L pravastatin for 5 h (n = 5). F-G. The concentrations of sFlt-1 (F) and sEng (G) were determined in samples collected from placental explants incubated in the absence or presence of pravastatin (Pra; 1, 10, 200 or 2000 micromol/L) for 72 h (n = 5). E-G. The concentrations were normalized to milligram of cultured tissue and expressed relative to Cntl (untreated cultures).

The concentrations of PlGF, sFlt-1 and sEng increased gradually with time of perfusion. Pravastatin in the maternal reservoir (PraM) did not significantly affect the concentration of PlGF (3.26±0.43 and 4.25±0.68 pg/g cotyledon for Cntl and PraM, respectively; Fig 1A), sFlt-1 (627.97±176.27 and 536.04±112.91 pg/g cotyledon for Cntl and PraM, respectively; Fig 1B) and sEng (46.29±11.39 and 36.41±5.86 pg/g cotyledon for Cntl and PraM, respectively; Fig 1C) measured at 5 h of perfusion compared to untreated (Cntl) cotyledons. VEGF could not be detected in samples collected from the maternal and fetal reservoirs.

#### Fetal circulation

We were unable to detect measurable concentrations of PlGF and sEng in the fetal reservoir. The concentrations of sFlt-1 measured (Fig 1D) were significantly lower compared to the concentrations measured in the maternal reservoir (Fig 1B). By 5 h of perfusion, similar concentrations of sFlt-1 were detected in Cntl (8.80±2.45 pg/g cotyledon) and PraM cotyledons (5.80±1.68 pg/g cotyledon) (Fig 1D).

Cultures of placental explants were treated with 0.2 micromol/L pravastatin for 5 h. Similar to the perfused cotyledons, the concentrations of sFlt-1 were not significantly affected by pravastatin (Fig 1E). The concentrations of PlGF and sEng were below the detection limit. Culturing of normal placental explants with pravastatin for 72 h did not alter the concentrations of sFlt-1 (Fig 1F) and sEng (Fig 1G). PlGF was not detected in supernatants collected from these cultures probably due to the presence of high concentrations of sFlt-1, reaching up to 600 pg/ml/mg tissue (not shown).

### The effect of pravastatin on eNOS expression and activation

**Fig 2:**
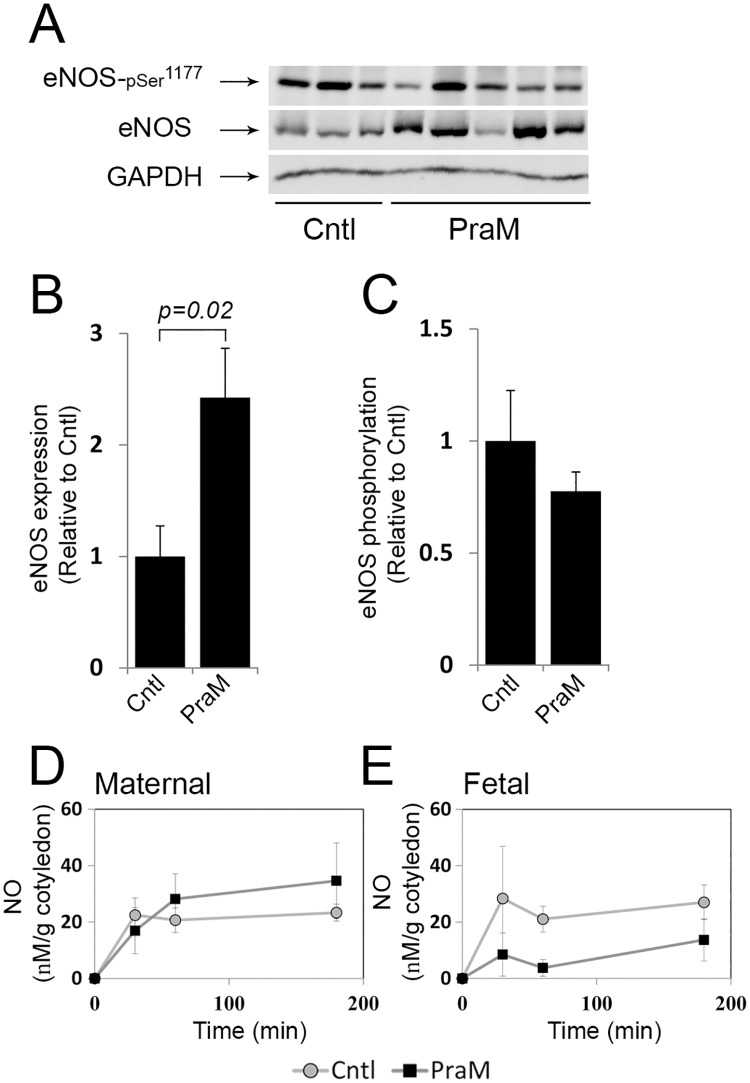
The effect of pravastatin on placental eNOS expression and activation in the human perfused cotyledon. Isolated cotyledons were perfused for 5 h in the absence (Cntl) or presence of 0.2 micromol/L pravastatin in the maternal (PraM) circulation (S1 Fig). A. a representative immunoblot demonstrating eNOS expression and activation, as detected by phosphorylation on serine 1177. B-C. The relative fold of eNOS and p-eNOS^Ser1177^ expression is shown by mean ± SEM. D-E. The concentration of NO in collected samples were determined by measurements of nitrite and normalized to gram of perfused cotyledon. Cntl: n = 6; PraM: n = 11.

The expression and activation of eNOS in perfused cotyledons in response to pravastatin are shown in Fig 2. As shown, pravastatin in the maternal reservoir (PraM) induced a significant increase in eNOS expression (Fig 2A–2B) compared to Cntl cotyledons. At 5 h of perfusion PraM did not significantly affect eNOS activation, detected by its phosphorylation (Fig 2A–2C). The concentrations of NO in the maternal reservoir (Fig 2D) and in the fetal reservoir (Fig 2E) did not significantly differ between Cntl and PraM cotyledons.

**Fig 3:**
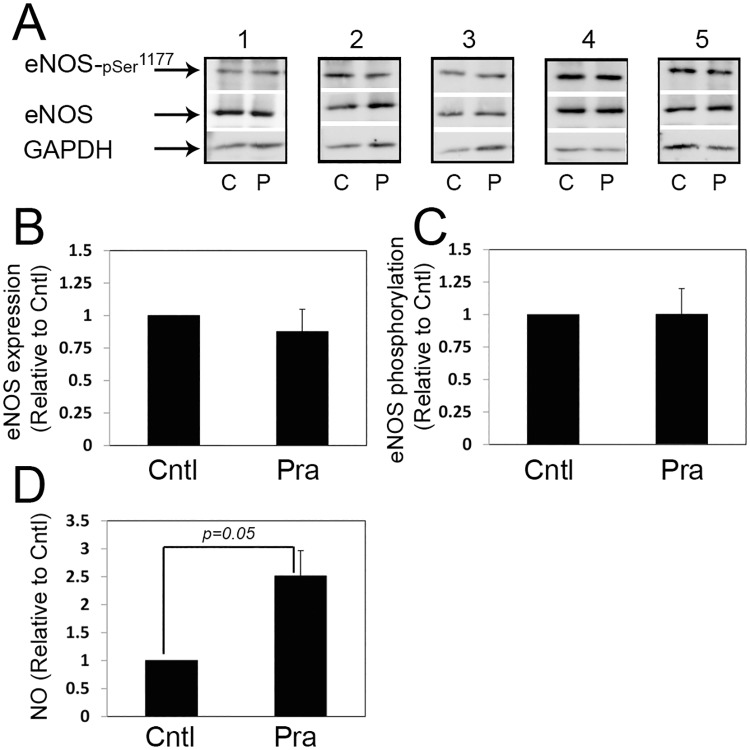
The effect of pravastatin on placental eNOS expression and activation in placental explant cultures. Placental explants were incubated in the absence (Cntl) or presence of 0.2 micromol/L pravastatin for 5 h. A. five immunoblots (corresponding to 5 different normal placentas) demonstrating eNOS expression and activation, as detected by phosphorylation on serine 1177; C, untreated cultures; P, pravastatin. B-C. The relative fold of eNOS and p-eNOS^Ser1177^ expression is shown by mean ± SEM. D. The concentration of NO in the culture medium were determined by measurements of nitrite and expressed relative to Cntl (untreated cultures). n = 5. Pra, pravastatin.

The expression and activation of eNOS were also analyzed in cultures of placental explants exposed to 0.2 micromol/L pravastatin for 5 h (Fig 3). As shown, in contrast to its effect in the perfused cotyledon, pravastatin did not affect eNOS expression in placental explants (Fig 3A and 3B). eNOS activity was also not affected (Fig 3A and 3C), however, pravastatin induced increased concentrations of NO compared to untreated explants (Cntl; Fig 3D).

**Fig 4:**
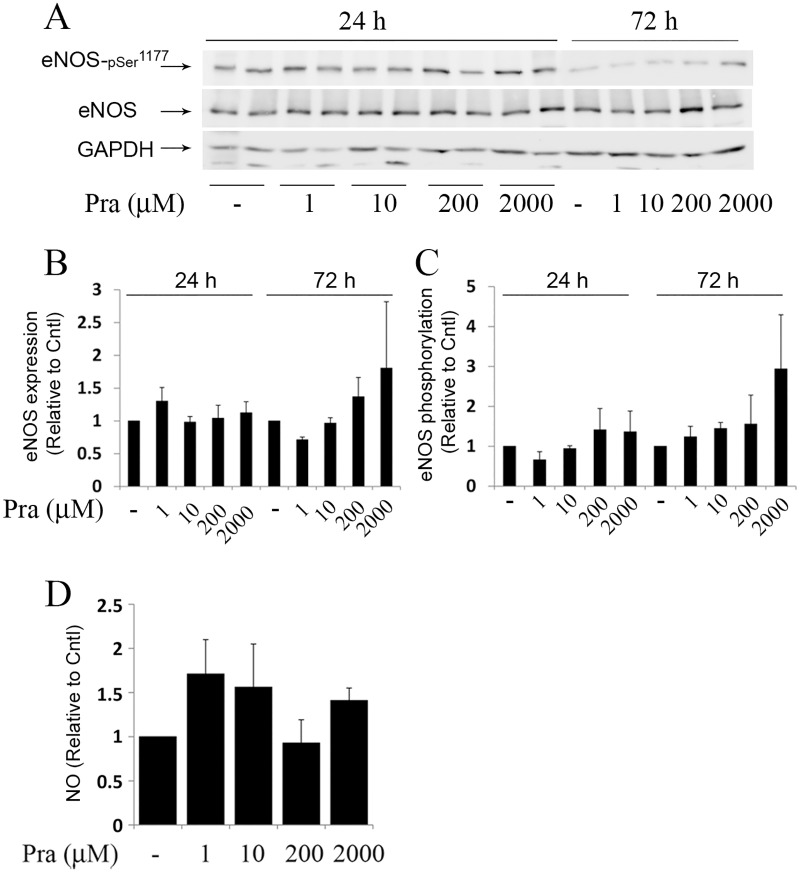
eNOS expression and activation in cultured placental explants exposed to various concentrations of pravastatin. Placental explants were incubated in the absence or presence of pravastatin (Pra; 1, 10, 200 or 2000 micromol/L) for 24 and 72 h (n = 5) (S2 Fig). A. a representative immunoblot demonstrating eNOS expression and activation, as detected by phosphorylation on serine 1177. B-C. The relative fold of eNOS and p-eNOS^Ser1177^ expression is shown by mean ± SEM. D. The concentration of NO in collected samples were determined by measurements of nitrite and expressed relative to Cntl (untreated cultures).

Exposure of placental explants to higher concentrations of pravastatin (1, 10, 200 or 2000 micromol/L) for 24 or 72 h, did not significantly change the expression (Fig 4A and 4B), activation (Fig 4A and 4C) of eNOS and the concentrations of NO in the culture medium (Fig 4D) compared to untreated cultures.

### The effect of pravastatin on fetal perfusion pressure

**Fig 5:**
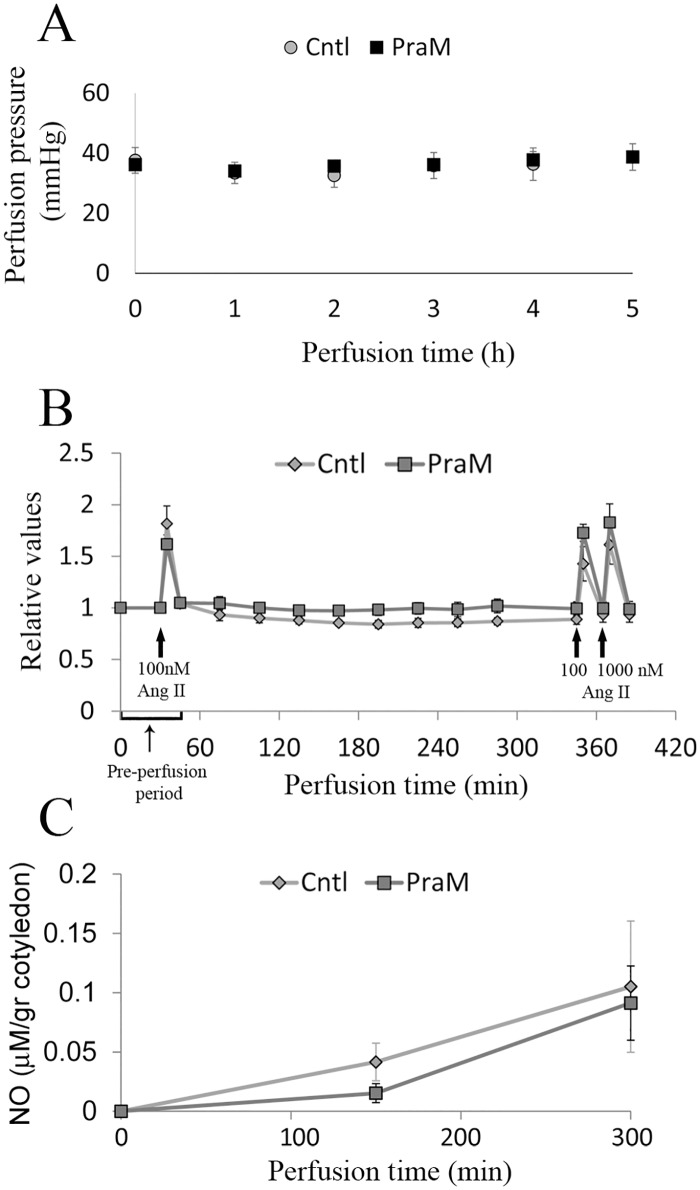
The effect of pravastatin on the feto-placental pressure. A. feto-placental pressure was monitored and recorded throughout the experiment and remained in the range of 25–40 mmHg. Cntl: n = 8; PraM: n = 11; B. Feto-placental vasoconstriction was induced by bolus injections of angiotensin-II as described in the "materials and methods" section. The cotyledons were allowed to recover between injections. Angiotensin-II-induced vasoconstriction was normalized to the baseline vasoconstriction and expressed as relative values of contraction. C. The concentrations of NO (normalized to gram of perfused cotyledon) in samples collected from the fetal reservoir following the experimental protocol as described in details in the "materials and methods" section. Cntl: n = 4; PraM: n = 3.

The fetal perfusion pressure in normal cotyledons perfused in the absence (Cntl) or presence of 0.2 micromol/L pravastatin in the maternal reservoir (PraM) is shown in Fig 5A. The fetal perfusion pressure monitored throughout the experiment was stable and remained within the range of 25–40 mmHg in Cntl and PraM cotyledons.

The effect of pravastatin on angiotensin-II-induced vasoconstriction is shown in Fig 5B. As shown, the fetal response to angiotensin-II injections was similar before and after perfusion in Cntl and PraM cotyledons. Accordingly, we did not detect any difference in the secretion of NO to the fetal circulation between Cntl and PraM cotyledons during the 5 h perfusion period (Fig 5C).

### The effect of pravastatin on placental eNOS expression and activation under reduced O_2_

**Fig 6:**
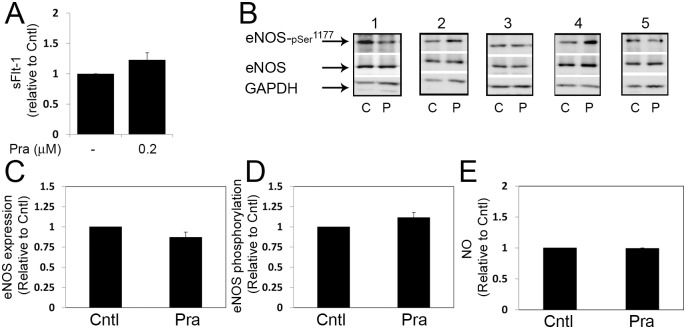
The effect of pravastatin on placental eNOS expression and activation under reduced O_2_ conditions. A. The effect of pravastatin on sFlt-1 secretion. Incubation for 5 h with pravastatin (0.2 micromol/L) did not affect sFlt-1 secretion from placental explants cultured under reduced O_2_ (hypoxia; 1% O_2_). Secreted protein levels were normalized against placental explant weight and expressed relative to untreated cultures. B. five immunoblots (corresponding to 5 different normal placentas) demonstrating eNOS expression and activation in untreated explants (C) and explants treated with 0.2 micromol/L pravastatin (P) for 5 h. C-D. The relative fold of eNOS and p-eNOS^Ser1177^ expression is shown by mean ± SEM. E. The concentration of NO in the culture medium were determined by measurements of nitrite and expressed relative to Cntl (untreated cultures). n = 5. Pra, pravastatin.

To determine whether pravastatin alters placental eNOS expression and activation under hypoxic conditions, placental explants were treated with 0.2 micromol/L pravastatin and cultured under reduced O_2_ (hypoxia; 1% O_2_) for 5 h (Fig 6). Pravastatin did not influence the concentrations of sFlt-1 in the culture medium (Fig 6A), the expression and activation of eNOS (Fig 6B–6D) and the concentrations of NO (Fig 6E) compared to untreated cultures.

**Fig 7:**
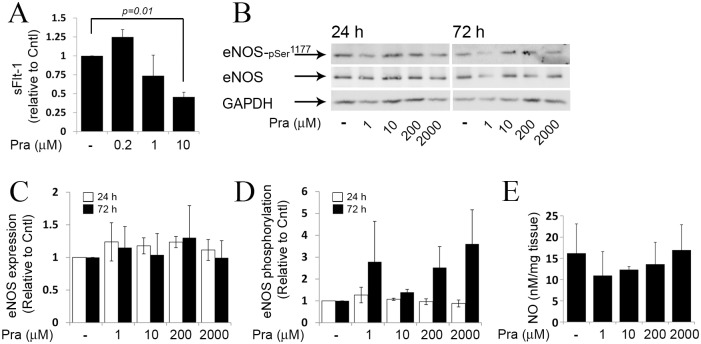
eNOS expression and activation in cultured placental explants exposed to various concentrations of pravastatin under reduced O_2_ conditions. A. The effect of pravastatin on sFlt-1 secretion. Pravastatin (10 micromol/L) reduced sFlt-1 secretion from placental explants cultured under reduced O_2_ (hypoxia; 1% O_2_). Secreted protein levels were normalized against placental explant weight and expressed relative to untreated cultures. B. Placental explants, cultured under reduced O_2_, were incubated in the absence or presence of pravastatin (Pra; 1, 10, 200 or 2000 micromol/L) for 24 and 72 h (n = 3) (S3 Fig). Representative immunoblots are shown, demonstrating eNOS expression and activation, as detected by phosphorylation on serine 1177. C-D. The relative fold of eNOS and p-eNOS^Ser1177^ expression is shown by mean ± SEM. E. The concentration of NO in collected samples were determined after 72 h of incubation by measurements of nitrite.

Treatment of placental explants with 10 micromol/L pravastatin for 72 h under hypoxic conditions reduced the secretion of sFlt-1 compared to untreated cultures (Fig 7A). Pravastatin treatment (1, 10, 200 or 2000 micromol/L) for 24 or 72 h under hypoxic conditions did not alter the expression and activation of eNOS (Fig 7B–7D) and the concentrations of NO in the culture medium measured after 72 h of culture (Fig 7E).

## Discussion

Preeclampsia is a major challenge to obstetricians. Despite extensive research effort, this disease is still incurable [2]. Encouraging results from animal studies have pointed at pravastatin as a possible novel effective treatment [15–18]. In humans, two small clinical trials highlight the potential of using pravastatin to treat or prevent preeclampsia; Costantine *et al*. have published novel preliminary safety and pharmacokinetic data regarding the use of pravastatin for preventing preeclampsia in high-risk pregnant women. They reported that the use of pravastatin was not associated with identifiable safety risks and provided evidence for possible efficacy (as evident from the lower rates of preeclampsia) [19]. Lefkou *et al*. have treated antiphospholipid antibody syndrome patients who developed preeclampsia and/or IUGR during thromboprophylactic treatment [20]. They have shown that pravastatin administration to these women may improve pregnancy outcomes when taken at the onset of preeclampsia or IUGR until the end of pregnancy. Most importantly, there were no congenital abnormalities or any fetal adverse effect because of the use of pravastatin.

Currently, data about the effect of pravastatin on the secretion of angiogenic factors from the normal placenta is limited to animal studies. In normal pregnancy of mice Kumasawa K *et al*. demonstrated increased PlGF levels [17], while Saad *et al* reported no change in sFlt-1 levels following treatment [18]. Our observation that pravastatin does not affect the secretion of sFlt-1 in the normal human placenta accords with the report from the study of Saad *et al*. The discrepancy between our data and the data of Kumasawa K *et al*. may result from differences between species and from differences in exposure time, as the mice in the study of Kumasawa K *et al*. were treated for 10 days [17].

Recent reports from the study of Kane *et al*. in sheep have raised considerable concern regarding the safety of using statins during pregnancy, due to the ability of pravastatin to attenuate the fetal peripheral vasoconstriction responses to acute hypoxic stress [32]. This effect was found to be mediated via increased NO bioavailability [32]. This valuable data should be interpreted carefully because pravastatin was injected directly to the fetus. Unlike fetal infusion, maternal treatment results in minimal exposure of the fetus [19]. Yet, the placenta, setting a barrier between the mother and the fetus, is exposed to the drug and the possibility that placental eNOS could be affected exists. This possibility may be important in light of the ability of NO to modify the feto-placental circulation [36].

Angiotensin-II is a vasoactive peptide that mediates NO-sensitive vasoconstriction [37, 38]. By using it, we were able to monitor the fetal vasoconstriction response that is sensitive to changes in NO bioactivity [37, 38] (Fig 5B). Our finding that the fetal vasoconstriction response to angiotensin-II, did not change following exposure of the maternal circulation to pravastatin accords with our observation that pravastatin does not alter the concentrations of NO in the fetal circulation (Fig 5C). These results are different from the results obtained by Kane *et al*. in the sheep model [32] and do not support the hypothesis that maternal treatment with pravastatin, attenuates the fetal vasoconstriction responses via NO changes. Further experiments in placental explants under normoxia (Fig 3) and reduced O_2_ conditions (Fig 6), expand our observations in the perfused cotyledon model, demonstrating that pravastatin treatment does not alter eNOS expression and activation. NO concentrations were not altered in response to pravastatin under hypoxia (Fig 6E), however, the concentrations were elevated under normoxia (Fig 3D). The changes in NO concentrations, between the two methods, following pravastatin treatment under normal conditions may result from differences in methodologies. Although the spatial relationships between the various cell types of the villous are retained in placental explants, the cotyledon model preserves the structural integrity of the placenta and most closely mimic the exposure of the different compartments of the placenta to the drug [33].

Animal studies play a key role in the development of new drugs. However, they cannot always be extrapolated to humans, especially because the placenta is the most species-specific mammalian organ [39, 40]. In the present study we used the *ex-vivo* human placental perfusion model and cultured placental explants. These experimental models allowed us to study several human placental functions in an organized placental tissue [41]. However, the present study has several limitations that should be acknowledged: 1. The *ex-vivo* human placental perfusion model may have limitations due to the short duration of the experiments. Still, studies have demonstrated that the pleotropic effects of statins can be detected within hours of treatment [26, 42]. Indeed, our findings demonstrating induced expression of eNOS within only a few hours of perfusion of the maternal side of the placenta with pravastatin, indicate that the pleotropic effects could be detected within the time frame of the study. In addition, by using placental explants we were able to investigate the pleotropic effects of pravastatin for up to 72 h. 2. In the present study, we used human placental tissue from normal pregnancy delivered at term. However, the use of pravastatin to prevent or treat preeclampsia may likely occur during early stages of gestation to term as evident from the clinical trials of Costantine *et al*. [19] and Lefkou *et al*. [20]. We cannot conclude that the response of the early placenta to the drug will be similar to that of the term placenta. Indeed, pravastatin treatment of first trimester trophoblasts limited the basal cell migration [43] and attenuated insulin-like growth factor actions, suggesting that it can impair placental cell turnover and fetal growth [44]. Thus, the results of the present study should be interpreted only to term pregnancy and may not reflect early gestation. 3. We cannot rule out the possibility that pravastatin will have different effect on the placental functions, investigated in the present study, under more physiological O_2_ concentrations (ambient 8% O_2_). Thus, further study on term and first trimester placental tissue under physiological O_2_ levels (corresponding to gestational stage) should add to our findings.

## Perspectives

Our findings demonstrate that treatment of normal human placenta with pravastatin does not change the basal placental physiology including metabolism and secretion of angiogenic factors. The treatment does not alter the feto-placental vascular tone or the fetal vasoconstriction response to angiotensin-II. Furthermore, pravastatin exposure does not alter eNOS expression, activation and NO concentrations at either normoxia or reduced O_2_ conditions. While other studies have marked the potential benefit of the treatment to the pathological placenta [16–18, 45], our results expand the current knowledge regarding the effect of the drug on the normal human placenta. The relevance of the study lays in the fact that treatment of high-risk patients with pravastatin may expose normal pregnancies to the drug.

## Supporting information

S1 Table Perfusion variables in 5 h perfusions. ^***a***^ Values are the mean ±SEM; M, Maternal reservoir; F, Fetal reservoir; NS, Not significant.
(DOCX)

S1 Fig eNOS expression and activation in the human perfused cotyledon. A-B. Isolated cotyledons were perfused for 5 h in the absence (Cntl) or presence of 0.2 micromol/L pravastatin in the maternal (PraM) circulation. Each number corresponds to a placenta.
(TIF)

S2 Fig eNOS expression and activation in cultured placental explants exposed to various concentrations of pravastatin. Placental explants were incubated in the absence or presence of pravastatin (Pra; 1, 10, 200 or 2000 micromol/L) for 24 and 72 h under normoxia; 21% O_2_ (n = 5). A-E Correspond to five different placentas.
(TIF)

S3 Fig eNOS expression and activation in cultured placental explants exposed to various concentrations of pravastatin under reduced O_2_ conditions. Placental explants, cultured under hypoxic conditions; 1% O_2_, were incubated in the absence or presence of pravastatin (Pra; 1, 10, 200 or 2000 micromol/L) for 24 and 72 h (n = 3). A-C Correspond to three different placentas.
(TIF)
