# Supplementary material for: The effects of pravastatin on the normal human placenta: Lessons from ex-vivo models
Source: PLoS One. 2017 Feb 15;12(2):e0172174. doi: 10.1371/journal.pone.0172174 (PMC5310776; doi:10.1371/journal.pone.0172174)
Supplement: S1 Table — a Values are the mean ±SEM; M, Maternal reservoir; F, Fetal reservoir; NS, Not significant. (DOCX) [file pone.0172174.s001.docx]

**S1 Table. Perfusion variables in 5 h perfusions.**

| **Characteristics** | **Cntl** | **PraM** | **P value** |
| --- | --- | --- | --- |
| **Glucose (mmol/L)***^a^* | M:8.28±0.40  F:9.66±0.68 | M:8.31±0.33  F:8.60±0.14 | NS |
| **Lactate (mmol/L)***^a^* | M:1.78±0.72  F:0.96±0.43 | M:1.83±0.30  F:1.38±0.15 | NS |
| **Fetal perfusion pressure (mmHg)** | 25-40 | 25-40 | NS |

*^a^*Values are the mean ±SEM; M, Maternal reservoir; F, Fetal reservoir; NS, Not significant.
