# Supplementary figures and images for: The effects of pravastatin on the normal human placenta: Lessons from ex-vivo models
Source: PLoS One. 2017 Feb 15;12(2):e0172174. doi: 10.1371/journal.pone.0172174 (PMC5310776; doi:10.1371/journal.pone.0172174)

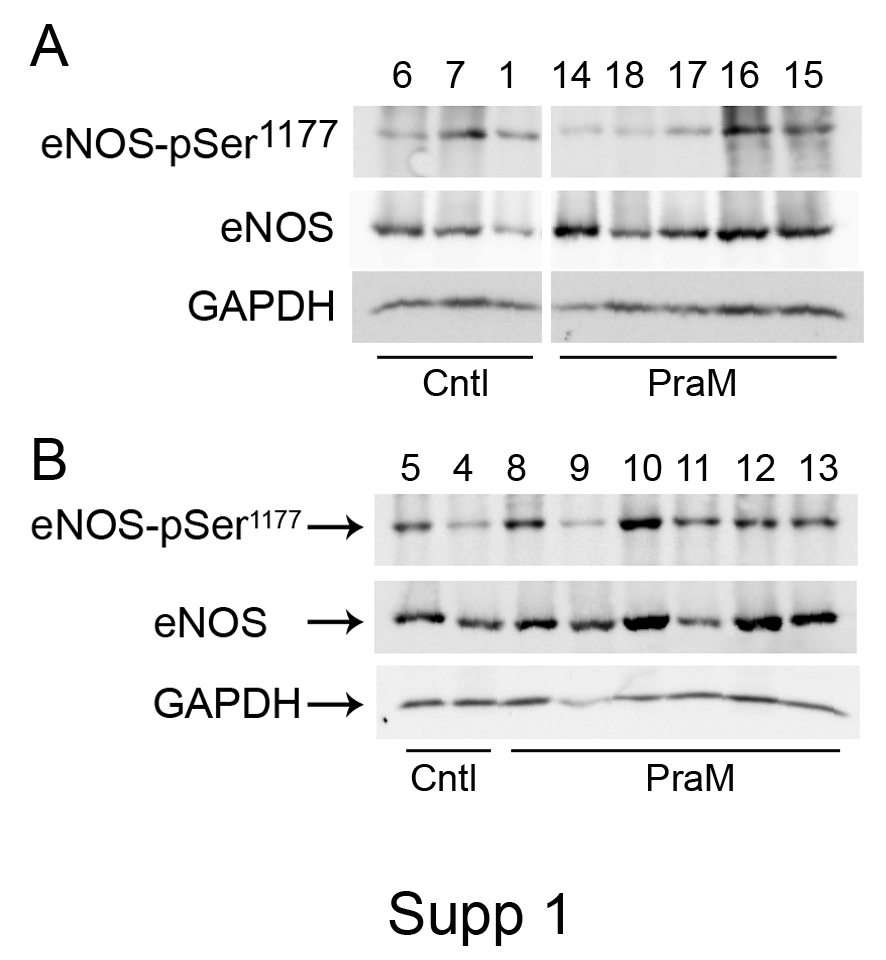

Supplement: S1 Fig — A-B. Isolated cotyledons were perfused for 5 h in the absence (Cntl) or presence of 0.2 micromol/L pravastatin in the maternal (PraM) circulation. Each number corresponds to a placenta. (TIF) [file pone.0172174.s002.tif]

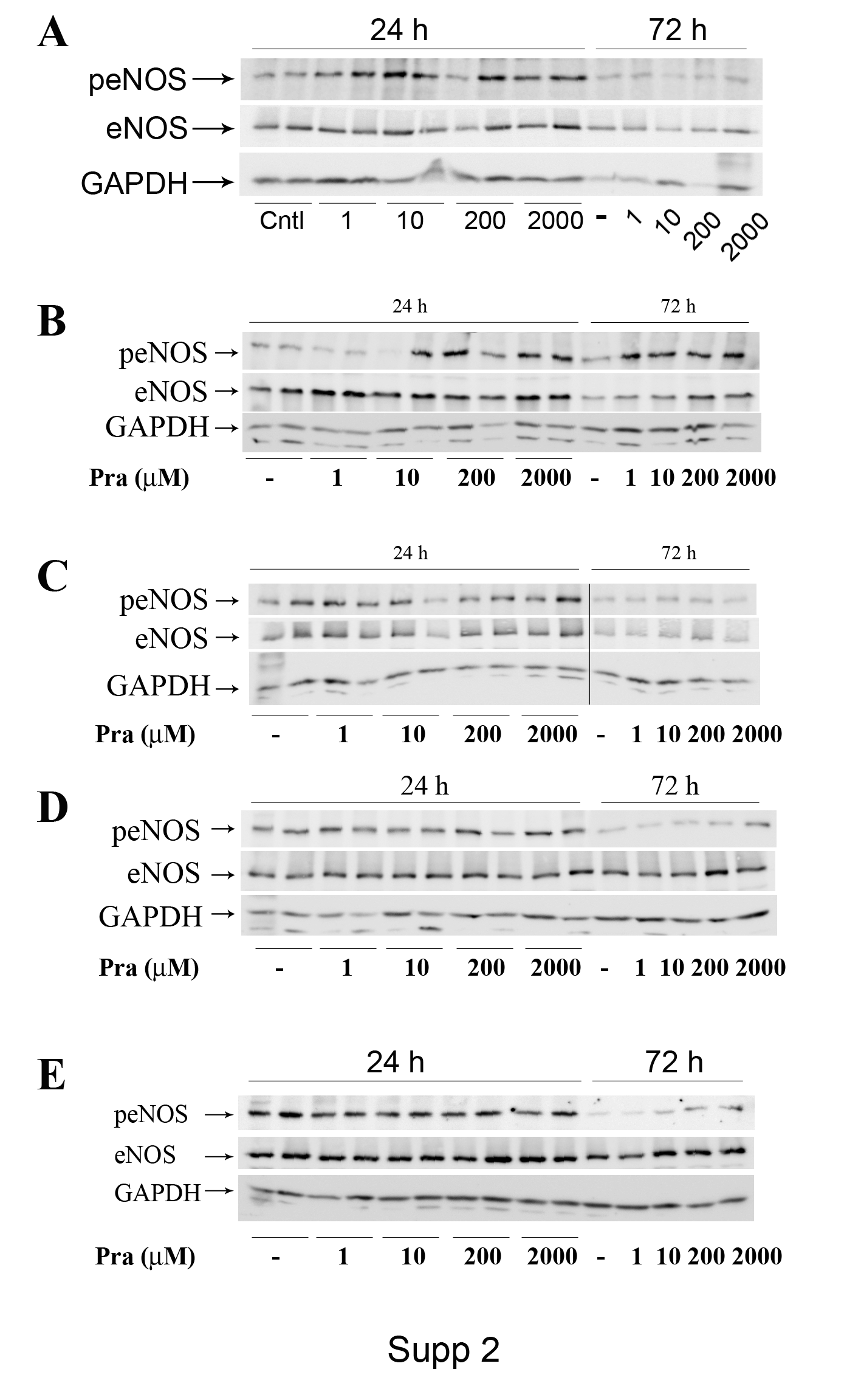

Supplement: S2 Fig — Placental explants were incubated in the absence or presence of pravastatin (Pra; 1, 10, 200 or 2000 micromol/L) for 24 and 72 h under normoxia; 21% O2 (n = 5). A-E Correspond to five different placentas. (TIF) [file pone.0172174.s003.tif]

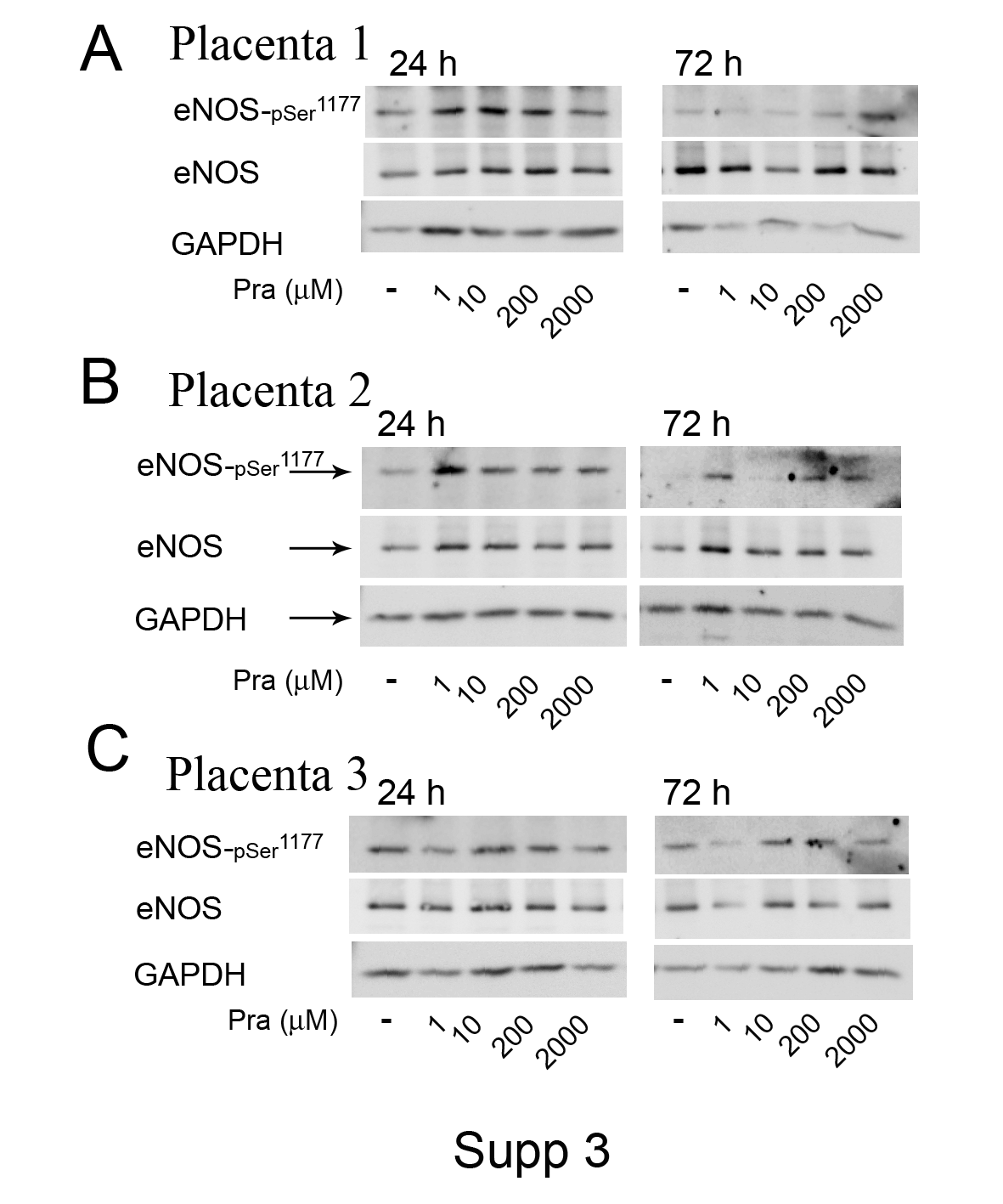

Supplement: S3 Fig — Placental explants, cultured under hypoxic conditions; 1% O2, were incubated in the absence or presence of pravastatin (Pra; 1, 10, 200 or 2000 micromol/L) for 24 and 72 h (n = 3). A-C Correspond to three different placentas. (TIF) [file pone.0172174.s004.tif]
